# Evaluation of retinal microcirculation alterations using optical coherence tomography angiography in patients with hyperopia ametropic amblyopia: A case-control study

**DOI:** 10.1097/MD.0000000000033196

**Published:** 2023-03-10

**Authors:** Ting Rao, Wen Zou, Xiaoqin Hu, Hai He, Wei Luo, Zhipeng You

**Affiliations:** a Affiliated Eye Hospital of Nanchang University, Nanchang, China; b Nanchang Hongdu Hospital of Traditional Chinese Medicine, Nanchang, China.

**Keywords:** foveal avascular zone, hyperopia ametropic amblyopia, optical coherence tomography angiography, perfusion density, vessel density

## Abstract

Given that there are controversial findings regarding vessel density in amblyopia, we quantified retinal microcirculation using optical coherence tomography angiography and compared it between hyperopic ametropic amblyopia eyes and age-matched control eyes. This case-control study was conducted from March 2021 to March 2022 at the Affiliated Eye Hospital of Nanchang University, Nanchang, China. Both groups included 72 eyes. Foveal avascular zone area, circularity and perimeter, perfusion density and vessel density of macular superficial retinal capillary plexus, macular thickness, macular volume, peripapillary retinal nerve fiber layer thickness, and ganglion cell-inner plexiform layer thickness were compared between hyperopia ametropic amblyopia eyes and age-matched control eyes. Additionally, best-corrected visual acuity, maximum corneal curvature, minimum corneal curvature, and anterior chamber depth were measured. In the hyperopia ametropic amblyopia eyes and control eyes, vessel density was 7.51 ± 2.13 and 9.91 ± 2.71 mm^-1^ in the central, 17.20 ± 1.38 and 18.25 ± 1.37 mm^-1^ in the inner, and 17.90 ± 0.88 and 18.43 ± 0.97 mm^-1^ in the full regions, respectively. The perfusion densities were 0.17 ± 0.06 and 0.23 ± 0.07 in the central, 0.41 ± 0.05 and 0.44 ± 0.03 in the inner, and 0.44 ± 0.03 and 0.46 ± 0.02 in the full regions, respectively. The central macular thicknesses of hyperopia ametropic amblyopia and control eyes were 240.04 ± 20.11 and 235.08 ± 24.41 µm, respectively. Foveal avascular zone perimeter and circularity (*P* < .043 and *P* = .001) significantly differed between the 2 groups. Hyperopia ametropic amblyopia eyes showed lower appreciably in vessel and perfusion densities, which could be one of the major pathophysiological mechanisms of hyperopia ametropic amblyopia and provide a new direction for the diagnosis and treatment of amblyopia.

## 1. Introduction

Amblyopia is a disease caused by strabismus, anisometropia, ametropia, and form deprivation, which leads to abnormal visual development. The monocular and binocular best-corrected visual acuity (BCVA) is lower than the normal level at the corresponding age. The affected patient does not have any organic lesions during an eye examination, but the BCVA of the 2 eyes differs by 2 or more lines, of which the worse eye is suggested to have amblyopia.^[1]^ The incidence of amblyopia is 0.8% to 3.3%.^[2]^ Because it seriously affects the visual development of children, it has become a key disease concern for amblyopia experts. The mechanism of amblyopia formation is very complex. Amblyopia pathogenesis is explained by 2 proposed theories: the central theory and the peripheral theory. In the peripheral theory, the retinal mechanism has always been the focus of visual research, although debatable.^[3–5]^ Thus far, the studies on the abnormal retinal structure and its mechanism in amblyopia are inconclusive.

Optical coherence tomography angiography (OCTA), a new vascular imaging technology developed based on optical coherence tomography (OCT), provides a detailed view of the microvascular networks for accurate, rapid, and noninvasive quantification.^[6]^ There have been many studies on the changes in the retinal microvascular system, measured using OCTA, related to human amblyopia.^[7–11]^ Many studies have confirmed that the vessel density (VD) of amblyopia macular in amblyopia eyes is lower than that in control eyes,^[7,9–11]^ yet other studies reported no change in VD.^[7,8]^ Some studies had included different types of amblyopia patients for observation and research, resulting in inconsistent research results between them.^[9,12]^

Because of the contradictory findings and unrecognized role of retinal-level amblyopia, we wanted to understand the relevant parameters of the retinal vascular system of hyperopia ametropic amblyopia and analyze the correlation among the parameters. The present study aimed to compare retinal microcirculation between hyperopic ametropic amblyopia eyes and age-matched control eyes using OCTA.

## 2. Methods

### 2.1. Study design

This study was conducted from March 2021 to March 2022 at the Affiliated Eye Hospital of Nanchang University. Patients with hyperopia ametropic amblyopia (approximately 4–12 years old) who were treated and observed on follow-up in the hospital during the study period were recruited. Additionally, the age-matched healthy children with normal vision were included as the normal control group. Among them, 72 eyes of 36 cases had hyperopia ametropic amblyopia, and 72 eyes of 36 control cases were controls. Hyperopia ametropic amblyopia was defined as binocular amblyopia combined with hyperopia ametropia. The difference in the spherical lens diopter was < 1.5 day, and that in the cylindrical lens diopter was < 1.00 day; refractory errors were converted into spherical equivalents (SE). Patients who were uncooperative or had mixed amblyopia, mental retardation, and any ocular or systemic abnormalities were excluded from the study.

This study followed the principles of the Declaration of Helsinki. Written informed consent was obtained for the guardians of the participants. This study was approved by the Ethics Committee of the Affiliated Eye Hospital of Nanchang University (YLP202012005).

### 2.2. Study methods

**Figure 1.**
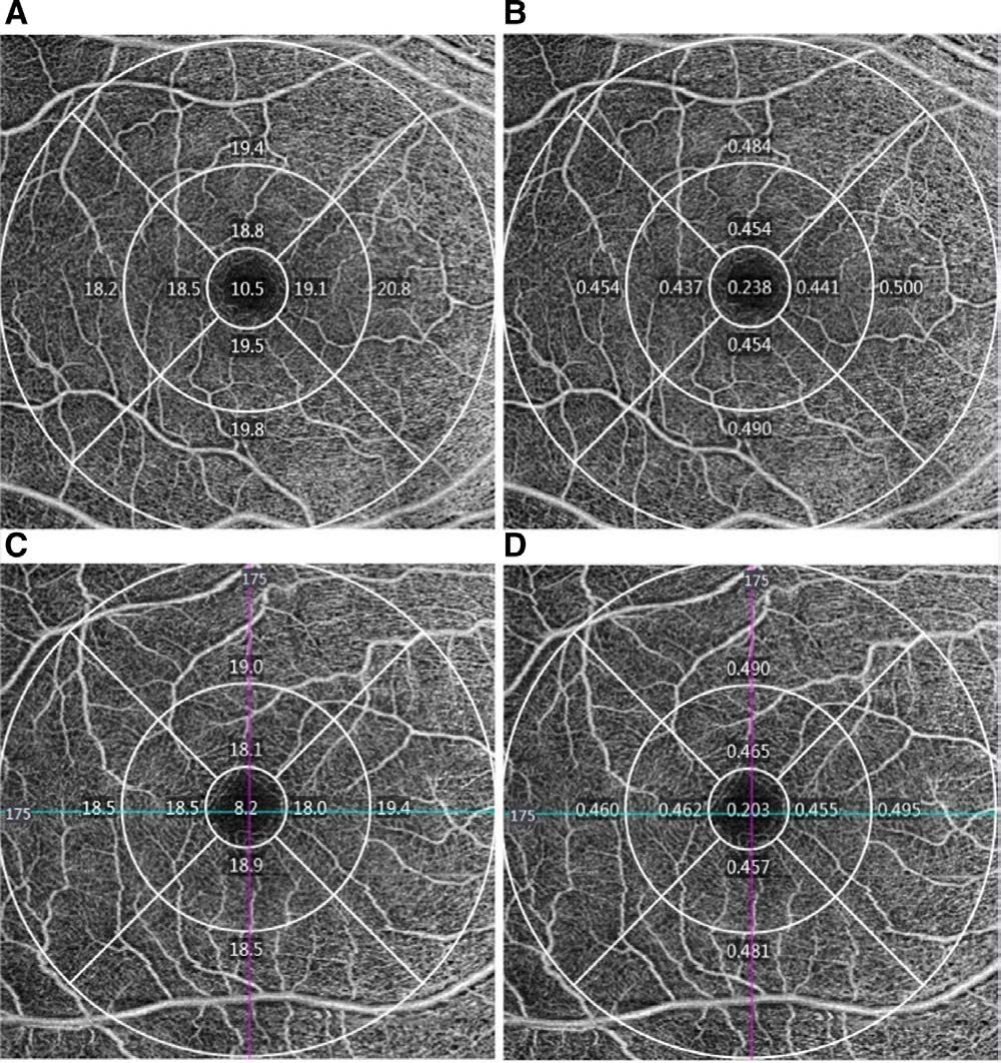
Optical coherence tomography angiography samples of hyperopia ametropic amblyopia and age-matched control. A: The vessel density in age-matched control eyes. B: The perfusion density in age-matched control eyes. C: The vessel density in hyperopia ametropic amblyopia eyes. D: The perfusion density in hyperopia ametropic amblyopia eyes.

All the subjects were examined for BCVA, cycloplegic refraction, eye position, slit-lamp, fundus photography, extraocular movements, axial length (AL), corneal curvature, and anterior chamber depth (IOL Master5.5; Carl Zeiss Meditec AG, Jena, Germany). Further, 6 mm × 6 mm imaging mode was used to scan the macular retina of each subject’s eyes using 5000-HD-OCT Angioplex (Carl Zeiss, Meditec, Inc., Dublin, OH). The system integrates retinal tracking technology to track and compensate eye movements in real time and combines optical microvascular complex algorithms to form a highly sensitive image. In this study, the shallow retina, namely the retina from the inner plexus layer to the inner limiting membrane layer, was used as the observation object. The OCTA software system was used to quantifiably analyze the digital blood flow information automatically in the macular region, and the VD and perfusion density (PD) of retinal blood vessels in the macular region (central region, inner region, outer region, and full region) were obtained. Besides, the inner and outer regions are equally divided into 4 regions: superior, nasal, inferior, and temporal (Fig. 1). Foveal avascular zone (FAZ) area (mm^2^), perimeter (mm), and circularity were obtained automatically. The exclusion criteria for OCTA examination were images with signal strength < 7 and severe motion artifacts due to poor fixation. All OCTA examinations were performed by the same experienced technician between 9:00 AM and 12:00 AM.

### 2.3. Statistical analysis

The normality of variables was tested by the Shapiro–Wilk test. Continuous variables are expressed as means ± standard deviations or medians (p25, p75), and categorical variables are expressed as numbers and percentages. The Welch 2-sample *t* test was used for variables conforming to a normal distribution, and Wilcoxon signed-rank test was used for variables not conforming to a normal distribution. A 1-way analysis of covariance, which was controlled using AL, was used to evaluate differences in the OCT and OCTA parameters. Spearman rank correlation analysis was used to explore the relationship among BCVA, SE, AL, VD, PD, FAZ area, perimeter, and circularity. *P* < .05 was considered statistically significant. SPSS 25.0 was used for statistical analysis (IBM, Armonk, NY).

## 3. Results

### 3.1. Participant baseline characteristics

Age (*P* = .476), sex (*P* = .346), K1 (*P* = .185), and K2 (*P* = .302) showed no significant difference between the cases and controls (Table 1). However, LogMAR-converted BCVA was significantly worse in hyperopia ametropic amblyopia cases compared to that in control cases (0.3 vs 0.0, *P* < .001). The SE, AL, and anterior chamber depth also differed significantly differed between the cases and controls, as shown in Table 1 (all *P* < .001) (Table 1).

**Table 1:** Basic information of research participants.

	Hyperopia ametropic amblyopia	Control	*P* value
Age (yr)	7.00 (5.00, 8.00)	7.00 (5.00, 9.00)	.476^a^
Sex (n, %)			
Male	16.00 (44.40)	20.00 (55.60)	.346^c^
Female	20.00 (55.60)	16.00 (44.40)	
BCVA (LogMAR)	0.30 (0.20, 0.50)	0.00 (0.00, 0.00)	<.001^a^
SE (D)	5.63 (3.50, 8.88)	0.25 (0.00, 0.50)	<.001^a^
AL (mm)	21.20 (20.23, 21.68)	22.92 (22.52, 23.75)	<.001^a^
K1 (D)	42.32 ± 1.61	42.67 ± 1.47	.185^b^
K2 (D)	43.52 ± 1.77	43.82 ± 1.70	.302^b^
ACD (mm)	3.19 (3.05, 3.36)	3.41 (3.19, 3.68)	<.001^a^

ACD = anterior chamber depth, AL = axial length, BCVA = best-corrected visual acuity, K1 = minimum corneal curvature, K2 = maximum corneal curvature, SE = spherical equivalent.

a Wilcoxon signed-rank test;

b Welch 2-sample *t* test;

c Chi-square test.

### 3.2. Macular thickness, retinal nerve fiber layer thickness, and ganglion cell-inner plexiform layer thickness

The central macular thickness (CMT) in hyperopia ametropic amblyopia eyes and control eyes was 240.04 ± 20.11μm and 235.08 ± 24.41 μm, respectively. After adjustment for AL, the CMT, average macular thickness, macular volume, early treatment diabetic retinopathy study (ETDRS) outer ring thicknesses in all regions, and ETDRS inner ring thicknesses (superior, nasal, inferior, and temporal) showed no statistically significant differences between the 2 groups (all *P >* .05) (Table 2).

**Table 2:** Comparison of macular thickness between hyperopia ametropic amblyopia eyes and control eyes.

	Hyperopia ametropic amblyopia	Control	*P* value^a^
CMT (µm)	240.04 ± 20.11	235.08 ± 24.41	.086
MV (mm³)	10.41 ± 0.62	10.20 ± 0.40	.770
AMT (µm)	289.60 ± 17.45	283.21 ± 11.36	.624
**ETDRS inner ring thickness** (µm)			
Superior	314.65 ± 24.87	317.71 ± 18.34	.211
Nasal	317.69 ± 22.90	319.24 ± 15.14	.209
Inferior	311.13 ± 24.62	312.33 ± 18.23	.391
Temporal	301.63 ± 21.16	306.56 ± 13.86	.765
**ETDRS outer ring thickness** (µm)			
Superior	298.97 ± 20.73	288.89 ± 15.27	.353
Nasal	309.58 ± 22.87	300.24 ± 17.44	.659
Inferior	283.99 ± 19.95	272.93 ± 13.64	.611
Temporal	276.32 ± 16.60	269.81 ± 15.76	.477

AMT = average macular thickness, CMT = central macular thickness, ETDRS = Early Treatment Diabetic Retinopathy Study, MV = macular volume.

a A 1-way analysis of covariance (ANCOVA).

Similarly, after adjusting for AL, the average values for retinal nerve fiber layer thickness (RNFLT) and RNFLT in all regions showed no statistically significant differences between the 2 groups (all *P* > .05). In addition, the ganglion cell-inner plexiform layer thickness had no significant difference in the average, minimum, and all regions between the 2 groups (Table 3).

**Table 3:** Comparison of ganglion cell-inner plexiform layer thickness and peripapillary retinal nerve fiber layer thickness between hyperopia ametropic amblyopia eyes and control eyes.

	Hyperopia ametropic amblyopia	Control	*P* value^a^
**RNFLT (µm**)			
Average	117.49 ± 22.42	104.17 ± 11.61	.262
Superior	143.82 ± 25.62	135.51 ± 23.15	.210
Nasal	79.01 ± 15.53	70.28 ± 11.73	.540
Inferior	162.38 ± 43.72	138.15 ± 19.73	.160
Temporal	84.90 ± 19.31	74.25 ± 10.49	.475
**GCIPLT (µm**)			
Average	85.03 ± 10.63	86.33 ± 5.79	.394
Minimum	75.50 ± 15.56	80.00 ± 11.40	.521
Superior	85.50 ± 12.97	86.90 ± 9.07	.505
Superonasal	87.99 ± 12.34	89.29 ± 7.46	.480
Inferonasal	86.33 ± 12.27	86.81 ± 5.56	.268
Inferior	82.32 ± 13.69	83.46 ± 7.72	.427
Inferotemporal	84.21 ± 9.00	85.69 ± 6.13	.117
Superotemporal	83.24 ± 10.77	85.67 ± 5.88	.936

GCIPLT = ganglion cell-inner plexiform layer thickness, RNFLT = peripapillary retinal nerve fiber layer thickness.

a A 1-way analysis of covariance (ANCOVA).

### 3.3. Vessel density, perfusion density, and foveal avascular zone

After adjustment for AL, the VD in hyperopia ametropic amblyopia eyes was significantly lower than that in the control eyes in the central (*P* < .001), inner (*P* < .001), full (*P* = .003), inner superior (*P* = .003), inner nasal (*P* = .001), inner inferior (*P* < .001), and inner temporal (*P =* .004) regions. The PD of macular superficial retinal capillary plexus in hyperopia ametropic amblyopia eyes was significantly lower than that in the control eyes in the central (*P* < .001), inner (*P* = .001), full (*P* = .015), inner superior (*P* = .001), inner inferior (*P* < .001), inner nasal (*P* = .016), inner temporal (*P* = .019), and outer superior (*P* = .036) regions. The FAZ perimeter (*P* = .043) and circularity (*P* = .001) significantly differed between the 2 groups. No significant differences were observed in VD in the outer region (*P* = .204) and in PD in the outer (*P* = .198), outer nasal (*P* = .657), outer inferior (*P* = .323), and outer temporal (*P* = .147) regions between the 2 groups (Table 4).

**Table 4:** Comparison of optical coherence tomography angiography findings between hyperopia ametropic amblyopia eyes and control eyes.

	Hyperopia ametropic amblyopia	Control	*P* value^a^
**VD (mm^-1^**)			
Central	7.51 ± 2.13	9.91 ± 2.71	<.001
Inner	17.20 ± 1.38	18.25 ± 1.37	<.001
Outer	18.49 ± 0.92	18.80 ± 0.92	.204
Full	17.90 ± 0.88	18.43 ± 0.97	.003
Inner superior	16.82 ± 2.69	18.32 ± 1.57	.003
Inner nasal	17.62 ± 1.43	18.28 ± 1.40	.001
Inner inferior	17.10 ± 1.77	18.29 ± 1.66	<.001
Inner temporal	17.10 ± 1.89	18.17 ± 1.56	.004
Outer superior	18.52 ± 1.29	18.90 ± 0.91	.125
Outer nasal	19.20 ± 1.44	19.76 ± 1.14	.149
Outer inferior	18.56 ± 1.52	18.93 ± 0.99	.102
Outer temporal	19.55 ± 18.77	17.48 ± 2.16	.517
**PD**			
Central	0.17 ± 0.06	0.23 ± 0.07	<.001
Inner	0.41 ± 0.05	0.44 ± 0.03	.001
Outer	0.46 ± 0.03	0.47 ± 0.02	.198
Full	0.44 ± 0.03	0.46 ± 0.02	.015
Inner superior	0.41 ± 0.07	0.44 ± 0.04	.001
Inner nasal	0.42 ± 0.05	0.43 ± 0.04	.016
Inner inferior	0.41 ± 0.06	0.44 ± 0.04	<.001
Inner temporal	0.41 ± 0.06	0.43 ± 0.04	.019
Outer superior	0.47 ± 0.03	0.48 ± 0.02	.036
Outer nasal	0.49 ± 0.03	0.49 ± 0.03	.657
Outer inferior	0.47 ± 0.03	0.48 ± 0.03	.323
Outer temporal	0.42 ± 0.06	0.44 ± 0.04	.147
**FAZ**			
Area (mm^2^)	0.32 ± 0.16	0.27 ± 0.09	.098
Perimeter (mm)	2.35 ± 0.67	2.07 ± 0.42	.043
Circularity	0.70 ± 0.11	0.76 ± 0.08	.001

FAZ = foveal avascular zone, OCTA = optical coherence tomography angiography, PD = perfusion density, VD = vessel density.

a A 1-way analysis of covariance (ANCOVA).

### 3.4. Correlation analysis

The BCVA and AL were significantly positively correlated with VD in the central (ρ = −0.371, *P* < .001 and ρ = 0.316, *P* < .001), inner (ρ = −0.472, *P* < .001 and ρ = 0.292, *P* < .001), outer (ρ = −0.249, *P* = .003 and ρ = 0.216, *P* = .009), and full (ρ = −0.362, *P* < .001 and ρ = 0.268, *P* = .001) regions; PD in the central (ρ = −0.349, *P* < .001 and ρ = 0.290, *P* < .001) region; and FAZ circularity (ρ = −0.229, *P* = .006 and ρ = 0.226, *P* = .006). In contrast, the SE was significantly negatively correlated with VD in the central (ρ = −0.381, *P* < .001), inner (ρ = −0.423, *P* < .001), outer (ρ = −0.194, *P* = .02), and full (ρ = −0.305, *P* < .001) regions and negatively correlated with PD in the central (ρ = −0.357, *P* < .001), inner (ρ = −0.281, *P* = .001), and full (ρ = −0.210, *P* = .011) regions and FAZ circularity (ρ = −0.265, *P* = .001). In addition, the BCVA and AL were significantly negatively correlated with the FAZ area (*P* = .049, *P* = .015) and perimeter (*P* = .010, *P* = .003); the SE was significantly positively correlated with the FAZ area (*P* = .038) and perimeter (*P* = .005) (Table 5).

**Table 5:** Correlation analysis among optical coherence tomography angiography findings, best-corrected visual acuity, spherical equivalent,and axial length.

	BCVA		SE		AL	
	ρ	*P* value^a^	ρ	*P* value^a^	ρ	*P* value^a^
**VD (mm**^−**1**^)						
Central	−0.371	<.001	−0.381	<.001	0.316	<.001
Inner	−0.472	<.001	−0.423	<.001	0.292	<.001
Outer	−0.249	.003	−0.194	.020	0.216	.009
Full	−0.362	<.001	−0.305	<.001	0.268	.001
**PD (mm**^−**1**^)						
Central	−0.349	<.001	−0.357	<.001	0.290	<.001
Inner	−0.327	<.001	−0.281	.001	0.113	.179
Outer	−0.097	.245	−0.049	.561	0.003	.968
Full	−0.257	.002	−0.210	.011	0.122	.146
**FAZ**						
Area (mm^2^)	0.165	.049	0.173	.038	−0.203	.015
Perimeter (mm)	0.214	.010	0.233	.005	−0.244	.003
Circularity	−0.229	.006	−0.265	.001	0.226	.006

AL = axial length, BCVA = best-corrected visual acuity, FAZ = foveal avascular zone, OCTA = optical coherence tomography angiography, PD = perfusion density, SE = spherical equivalent, VD = vessel density.

a Spearman rank correlation analysis.

## 4. Discussion

This study quantified retinal microcirculation using OCTA and compared it between hyperopic ametropic amblyopia eyes and age-matched control eyes. Regarding the angiographical findings, we noted a significant decrease in VD in the central, inner, full, and all the inner regions in hyperopia ametropic amblyopia eyes compared with control eyes. Furthermore, the PD in the central, inner, full, inner superior, inner nasal, inner inferior, inner temporal, and outer superior regions of hyperopia ametropic amblyopia eyes was significantly reduced. In the study, we found a reduction in FAZ circularity. Compared with the control eyes, the FAZ perimeter of hypermetropic ametropic amblyopia eyes expanded. According to the results of correlation analysis, these changes in VD, PD, and FAZ circularity were more significant with the curtailment of AL and BCVA. However, these alterations in VD, PD, and FAZ circularity negatively correlated with SE. These results are compatible with those reported in the recent literature, which showed a reduced VD on OCTA, although some studies used different regions of interest.^[13–15]^

In the current study, we observed no significant differences in the CMT, macular volume, average macular thickness, ETDRS outer ring thickness, and ETDRS inner ring thickness between the hyperopia ametropic amblyopia eyes and control eyes. Our results are consistent with those of published studies.^[16–18]^ However, previous studies showed that amblyopia eyes had thicker macular retinal thickness than normal eyes, with a significant difference between amblyopia eyes and normal eyes.^[5,19,20]^ This discrepancy could be due to differences in age, sex, and types of amblyopia of the study participants.^[5,16–20]^ Furthermore, RNFLT and ganglion cell-inner plexiform layer thickness in all regions were not different between the 2 groups, although RNFLT was thicker in hyperopia ametropic amblyopia eyes than in control eyes. Recently, Kasem et al^[5]^ reported that the CMT and RNFLT were thicker in amblyopic eyes than in fellow eyes. The apoptosis of postpartum retinal ganglion cells could be inhibited by amblyopia and show an increase in RNFLT, which was confirmed by Yen study findings.^[21]^ Kim Yong Woo study showed that the macular GCIPL thickness had no significant difference among the 3 groups.^[22]^ We believe that abnormal visual experience reduces the degeneration of retinal ganglion cell (RGC), thereby hindering the degeneration of the retina and the formation of the fovea. Our results are consistent with those reported in the literature.^[16,23,24]^

In the past, most of the studies on the microvascular changes of amblyopia focused on strabismic, anisometropic, or mixed amblyopia. The research object of this experiment was hyperopia ametropic amblyopia, avoiding the confounding factors occurring from the mixed amblyopia. Our study showed that VD, PD, and FAZ circularity decreased, indicating arrest of retinal microvascular development, obstruction of fundus vascularization, redistribution of blood flow, and potentially widened retinal vessels, respectively. The most interesting finding in this study is that VD and PD in hyperopia ametropic amblyopia eyes were significantly lower in the inner region, but not in the outer region. This finding could be because the outer region was closer to the retinal artery and choroidal circulations. Besides, the degradation of RGC function and the decrease in RGC numbers might reduce the demand for dense vascular system by the retina. The previous randomized controlled trials showed VD in amblyopia eyes was lower than that in the fellow eyes and control subjects’ eyes.^[24,25]^ Additionally, a comparative study was conducted on 30 amblyopia patients and 1045 control subjects in Hong Kong. The FAZ circularity and fractal dimension in amblyopic eyes were lower than those in control eyes.^[7]^ These findings are consistent with our study findings. However, Araki et al^[26]^ reported no significant difference in the VD between amblyopic eyes and nonamblyopic eyes after magnification error correction. Moreover, Araki et al^[26]^ and Demirayak et al^[8]^ found that the FAZ area was smaller in the amblyopic eyes than in the fellow eyes, whereas Sobral et al^[25]^ reported that the FAZ area of superficial retinal capillary plexus and DCP increased. In our study, we observed an increase in FAZ area and FAZ perimeter. The difference in the results between these studies may be due to the following reasons. First, because of different measuring instruments, there might be some differences between RTVue XR Avanti (Optovue Inc., Fremont, CA) and Zeiss Cirrus 5000-HD-OCT Angioplex (Carl Zeiss, Meditec, Inc., Dublin, OH) used in this test. Second, the type and severity of amblyopia (strabismic, anisometropic, or mixed amblyopia) might have influenced the data. The results of the correlation analysis helped us determine the positive association of VD and FAZ circularity with AL and BCVA and the negative association with SE, suggesting that abnormal visual stimulation hindered the development of retinal microvessels in the macular area of amblyopia and the formation of normal fundus vascular morphology. Our research showed that abnormal blood flow might be the pathogenesis of hyperopia ametropic amblyopia, suggesting that improving blood flow might become a new direction of amblyopia treatment, even though the mechanism of VD reduction in hyperopia ametropic amblyopia was unclear.

This study has some limitations. Our study lacked data on VD and PD of the macular deep retinal capillary plexus. Second, we did not dynamically observe whether the density of the macular deep retinal capillary plexus and the macular thickness of hyperopia ametropic amblyopia patients changed with improvement in vision. Third, the sample size we included was relatively small. Additionally, this study investigated only the difference in retinal microcirculation between hyperopia ametropic amblyopia and age-matched control. The comparisons of the degree of amblyopia related to the effect of retinal microcirculation warrants further research.

In conclusion, our study demonstrated that VD and PD decreased and FAZ circularity reduced in hyperopia ametropic amblyopia eyes. The VD and PD in the partial regions increased with greater AL and BCVA. A negative correlation was observed between SE and VD and PD. Based on our findings, we speculate that retinal vascular changes play an important role in the pathogenesis of hyperopia ametropic amblyopia. Thus, whether we can treat and prevent amblyopia by improving the patient’s blood flow is the subject of our next research.

## Acknowledgments

We would like to thank all participants of the study and their families. We would especially like to thank Jiangying Wang for assisting in participant testing and Guofu Wu for statistical information consultation.

## Author contributions

**Conceptualization:** Ting Rao, Xiaoqin Hu, Zhipeng You.

**Data curation:** Wen Zou, Hai He.

**Formal analysis:** Wen Zou.

**Validation:** Wen Zou, Hai He, Wei Luo.

**Investigation:** Xiaoqin Hu.

**Methodology:** Zhipeng You.

**Resources:** Hai He, Wei Luo.

**Supervision:** Zhipeng You.

**Writing – original draft:** Ting Rao, Zhipeng You.

**Writing – review & editing:** Ting Rao, Zhipeng You.
